# Lipid Profile and Hepatic Fat Content Measured by ^1^H MR Spectroscopy in Patients before and after Liver Transplantation

**DOI:** 10.3390/metabo11090625

**Published:** 2021-09-15

**Authors:** Martin Burian, Milan Hajek, Petr Sedivy, Irena Mikova, Pavel Trunecka, Monika Dezortova

**Affiliations:** 1 MR-Unit, Department of Diagnostic and Interventional Radiology, Institute for Clinical and Experimental Medicine, 140 21 Prague, Czech Republic; martin.burian@ikem.cz (M.B.); milan.hajek@ikem.cz (M.H.); petr.sedivy@ikem.cz (P.S.); 2 Department of Hepatogastroenterology, Institute for Clinical and Experimental Medicine, 140 21 Prague, Czech Republic; irena.mikova@ikem.cz (I.M.); pavel.trunecka@ikem.cz (P.T.)

**Keywords:** magnetic resonance, MR spectroscopy, liver, steatosis, lipid saturation, lipid profile, NASH, NAFLD, transplantation

## Abstract

Increased hepatic fat content (HFC) is a hallmark of non-alcoholic fatty liver (NAFL) disease, a common condition in liver transplant recipients. Proton MR spectroscopy (^1^H MRS) and MR imaging-based proton density fat fraction as the only diagnosis modality enable precise non-invasive measurement of HFC and, also, fatty acid profiles in vivo. Using ^1^H MRS at 3T, we examined 47 liver transplantation candidates and 101 liver graft recipients. A point-resolved spectroscopy sequence was used to calculate the steatosis grade along with the saturated, unsaturated and polyunsaturated fractions of fatty acids in the liver. The steatosis grade measured by MRS was compared with the histological steatosis grade. HFC, represented by fat fraction values, is adept at distinguishing non-alcoholic steatohepatitis (NASH), NAFL and non-steatotic liver transplant patients. Relative hepatic lipid saturation increases while unsaturation decreases in response to increased HFC. Additionally, relative hepatic lipid saturation increases while unsaturation and polyunsaturation both decrease in liver recipients with histologically proven post-transplant NASH or NAFL compared to non-steatotic patients. HFC, measured by in vivo ^1^H MRS, correlated well with histological results. ^1^H MRS is a simple and fast method for in vivo analysis of HFC and its composition. It provides non-invasive support for NAFL and NASH diagnoses.

## 1. Introduction

Non-alcoholic fatty liver disease (NAFLD), which results from excessive liver fat accumulation (steatosis), is one of the most frequent liver diseases and is characterized by varying degrees of progression. Conditions range from liver steatosis [1], which can progress in some patients to non-alcoholic steatohepatitis (NASH) [2] characterized by inflammation and hepatocyte ballooning, to fibrosis, cirrhosis and/or hepatocellular carcinoma [3].

Liver graft biopsy is the most comprehensive method for determining liver graft pathology including hepatic fat content (HFC). However, the small volume of liver biopsies performed is a limiting factor of HFC quantitative measurement, which supports the application of non-invasive imaging methods [4,5]. Two of the most effective methods for steatosis measurement are the magnetic resonance imaging technique known as proton density fat fraction (PDFF) and MR spectroscopy (MRS) calculation of fatty acid fractions (FF). Both methods are commonly used in clinical practice to quantify HFC in patients with various liver diseases and to evaluate the effects of certain treatments, dietary interventions or exercise on hepatic steatosis [6,7,8].

Magnetic resonance (MR) is a key instrument used in the examination of living liver donors before liver transplantation (LT) [9,10] as it minimizes the risks of liver biopsy [10,11,12,13]. However, studies focusing on LT recipients are rare [14].

**Figure 1:**
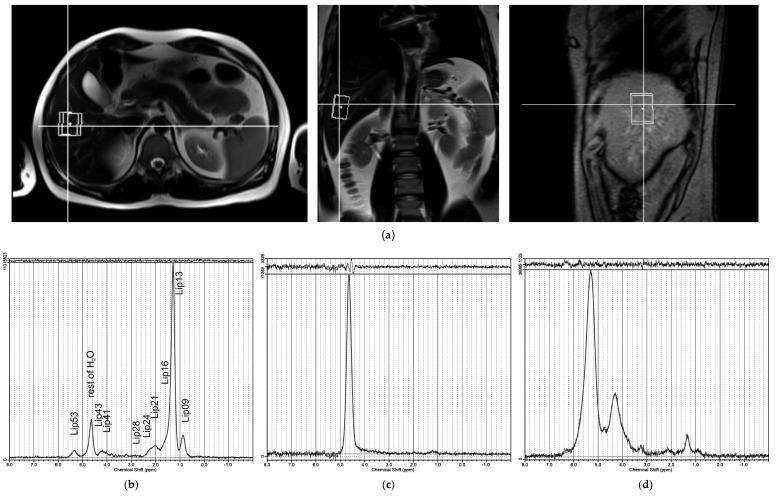
(**a**) Position of spectroscopic voxel (40 × 30 × 25 mm) in the liver for PRESS sequence (TE = 30 ms), with localization based on three orthogonal anatomical MR slices. Examples of LCModel [15] outputs of water-suppressed ^1^H MR spectra: (**b**) good spectrum quality with signals labelled as per Table 1; (**c**) spectrum excluded from further analysis due to low –(CH_2_)_n_– S/N ratio; (**d**) spectrum excluded due to poor shim or movement artifacts.

Well-resolved and high-resolution quantitative nuclear magnetic resonance (NMR) spectroscopy of fatty acids (FA) extracts measured at a magnetic field of 9.3T and higher allows up to 30 parameters describing FAs mixture to be calculated. Unfortunately, this is not the case with in vivo MRS of liver and other tissues. Due to low spectral resolution, it is not possible to characterize individual fatty acids but only hydrogen atoms in functional groups representing FA in the mixture. Thus, in vivo MR spectra measured between 1.5 and 7T only allow for the interpretation of approximately 10 groups of proton signals (Table 1, Figure 1).

Signal intensities can be used to calculate the fractions of hydrogen atoms in saturated (f_SI_), unsaturated (f_UI_) and polyunsaturated (f_PUI_) functional groups in FA chains. This so-called group or type analysis of liver or adipose tissue has been described in several papers and has shown that these results can be used as valuable supplementary data to PDFF and/or FF results [16,17,18,19,20,21].

The aims of our study were to conduct a detailed analysis of liver fat metabolic profiles using MRS and to calculate the fractions of saturated, monounsaturated and polyunsaturated functional groups in fatty acid chains in relation to the presence of steatosis/steatohepatitis in patients before and after LT.

## 2. Results

### 2.1. Steatosis Grade

Liver graft biopsy was available in 84 of 101 patients after liver transplantation (Tx group). Three patients refused biopsy and 14 patients were evaluated six months after Tx where biopsy was not part of the protocol. Most patients in the Tx group developed no (S0: 40.5%) or mild (S1: 40.5%) steatosis, with significant steatosis (S2: 10.7% or S3: 8.3%) only occurring in 19.0% of patients (Table 2).

Steatosis was graded using FF values calculated from MR spectra in correlation with histological grading as described in our previous study [14]. Based on MR grading, subjects were distributed in each Tx data set as follows: grade S0^mr^—21.8%; S1^mr^—61.4%; S2^mr^—8.9%; S3^mr^—7.9%. Altogether, 84 (83.2%) out of 101 Tx patients had no or mild steatosis grades S0^mr^ and S1^mr^ with corresponding low FF values. The remaining 17 subjects had steatosis grades S2^mr^ and S3^mr^ with liver fat content greater than 6.07% (in agreement with the HFC value in [14]), indicating significant steatosis. In the liver transplantation candidates (WL) group, MR grading showed 95.7% patients with no or mild steatosis (grade S0^mr^—34.0%; S1^mr^—61.7%) (Table 2).

**Figure 2:**
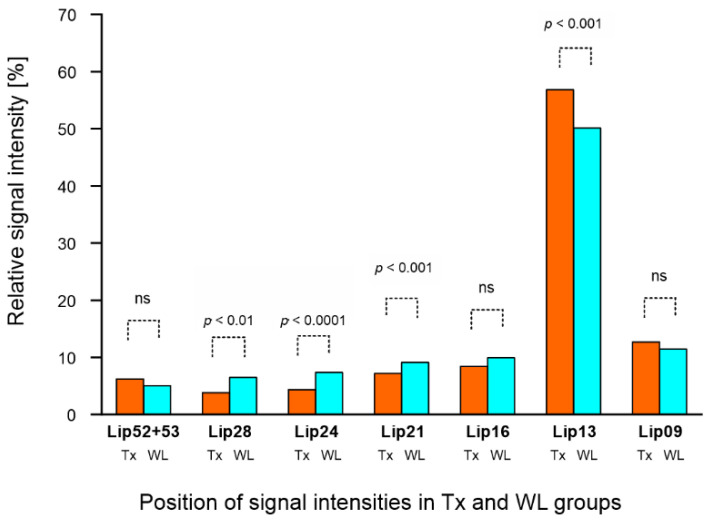
MR metabolic profiles of lipids in liver transplant recipient (Tx) and candidate (WL) groups independent of steatosis grade. Relative signal intensities (in [%]) of liver fatty acids were obtained from spectra with water suppression within chemical shift ranges of 0–3 ppm and 5.2–5.3 ppm.

In addition to the FF calculation, relative intensities of seven FA signals in liver spectra (FA metabolic profile) were compared based on LCM spectra results. Figure 2 shows a graphical representation of the differences in metabolic profiles between the Tx and WL groups independent of steatosis grade. There were significant differences between lipid signals Lip13, Lip21, Lip24 and Lip28.

### 2.2. Lipid Profile

**Figure 3:**
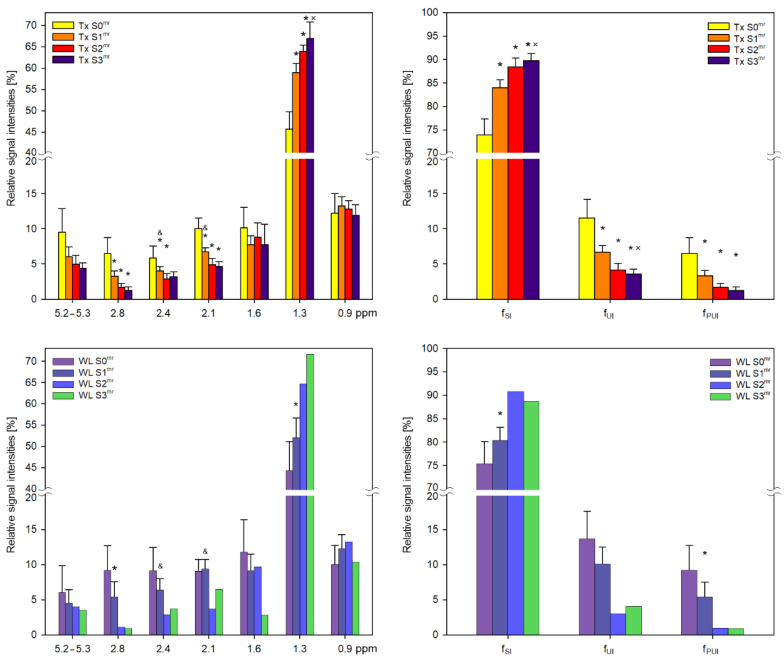
MR metabolic profiles of lipids in liver transplant recipient (Tx) and LT candidate (WL) groups with respect to steatosis grade. Relative signal intensities (in [%]) together with 95% intervals of confidence of liver fatty acids within chemical shift ranges of 0–3 ppm and 5.2–5.3 ppm were obtained from spectra with water suppression in Tx and WL groups. Fractions of hydrogen atoms [%] in saturated (f_SI_), unsaturated (f_UI_) and polyunsaturated (f_PUI_) fatty acid chains are also demonstrated. With regard to S2 and S3 WL cases, only data for one subject are shown for comparison purposes. * significant difference from S0^mr^; ^×^ significant difference from S1^mr^; ^&^ significant difference between Tx and WL groups.

In both groups of patients, we evaluated lipid profiles by calculating the relative signal intensities of the groups of liver fatty acid chains for each steatosis grade (S0^mr^–S3^mr^) (Figure 3). Multi-parametric comparison by ANOVA revealed significant differences between S0^mr^ and other steatosis grades in the intensities of aliphatic CH_2_ (Lip13, Lip24), allylic (Lip21) and diallylic (Lip28) signals. We observed significant changes in Lip13 signal intensities, indicating an increase in the proportions of saturated bonds in FA chains in correlation with increasing steatosis grade. Conversely, there was a significant decrease in the intensities of allyl and diallyl signals, indicating a reduction in unsaturated double bonds in correlation with increasing steatosis grade. Although olefinic signals also decreased in correlation with steatosis, S0^mr^ relative signal intensities were characterized by wide confidence intervals due to low signal strength and differences between groups were insignificant. Similar results were obtained for the WL group (Figure 3).

FA chain characteristics are semi-quantitatively classified according to f_SI_, f_UI_ and f_PUI_ and calculated according to Equations (3)–(5) (Figure 3). Fractions represent saturated, unsaturated and polyunsaturated hydrogen bonds of FA chains. There was also an increase in the proportion of hydrogen atoms in saturated bonds in correlation with steatosis and vice versa for unsaturated bonds.

Classification of post-Tx patients based on NAFLD activity score (NAS) and histological results (Table 3) revealed 34 non-steatosis patients, 39 patients with NAFL (non-alcoholic fatty liver) (NAS score 1–4) and 11 patients with NASH (NAS 5–8). The remaining 17 patients had no histology.

As with steatosis progression, FA saturation was higher while unsaturation and polyunsaturation were both lower in LT recipients with histologically proven post-transplant NASH or NAFL than in non-steatosis patients.

## 3. Discussion

MR imaging and spectroscopy are routinely used to determine liver fat content during clinical MR examinations. As previously reported in several studies, MRS facilitates analysis of MR spectra to describe fatty acid profiles in the liver [16,17]. To the best of our knowledge, this is the first study to use detailed in vivo ^1^H MRS for non-invasive assessment of hepatic lipid signal profiles in LT recipients and candidates.

In agreement with our previous findings [23], liver steatosis was highly frequent in LT recipients (78%) and LT candidates (66%). We observed a significant difference in hepatic FF content between LT recipients with histologically proven post-LT NASH, NAFL or non-steatosis. Relative hepatic lipid saturation increased while unsaturation decreased in correlation with increased HFC in liver grafts. Relative hepatic lipid saturation was significantly higher but unsaturation was lower in recipients with histologically proven post-LT NASH and NAFL than in non-steatosis patients. Polyunsaturation was only significantly different between NASH and non-steatosis recipients.

The first step of our data analysis was to compare the distribution of FF values between Tx and WL groups. As demonstrated in Table 2, FF reflected overall steatosis regardless of whether the liver was intrinsic or transplanted. It should be noted that patients with impaired liver function generally exhibit low grades of liver steatosis (only two out of forty-seven WL patients had S2^mr^ or S3^mr^), which is typically attributable to variations in liver tissue composition; fibrotic tissue gradually predominates with only a small amount of residual liver fat.

Our detailed spectra analysis revealed that in both Tx and WL groups, increased steatosis grade corresponded with a significant increase in the number of protons in saturated carbon bonds (increased Lip13 signal) and a decrease in the number of unsaturated bonds (Lip21 and Lip28 signals). These changes were also reflected in the saturation fractional indices for f_SI_, f_UI_ and f_PUI_.

Fractions of saturated hepatic FA bonds significantly increased, while fractions of unsaturated and polyunsaturated bonds decreased in patients with post-LT steatosis and histologically proven post-LT NASH. This finding reflects the acceleration of fatty acid metabolism, which is involved in the pathogenesis of NASH, even in LT patients.

Our results tally with other reports on human hepatic tissue that employed chromatography and other analytical in vitro methods. As far back as 50 years ago, Singer [24] showed that mono-, di- and polyunsaturated fatty acid content differs between normal and steatotic livers and that polyunsaturated fatty acid (PUFA) content decreases in correlation with increased diameter of fat droplets.

Those findings have been subsequently confirmed by several authors. Araya et al. [25] observed decreased PUFA and LCPUFA (long-chain PUFA) content and increased saturated hepatic fatty acid levels in NAFLD patients with simple steatosis or steatohepatitis compared to controls. They postulated that depletion of hepatic LCPUFA may result from both defective desaturation of PUFA and from increased peroxidation of LCPUFA due to oxidative stress. They also found that enhancement of the n-6/n-3 ratio indicates suppression of liver FA oxidation and secretion and that lipids deposited in the liver lead to steatosis.

Lipidomic analysis of patients with NASH, NAFL and controls revealed differences in total lipids between controls and patients as well as significant differences in mono- and polyunsaturated fatty acids and in the chemical composition of fatty acids [26]. Significant changes in total PUFA and total LCPUFA have also been observed in obese patients and controls [27]. These findings confirm NAFLD as a highly lipotoxic clinical condition. Experimental studies confirm that saturated free FA in circulation play an important role in hepatic lipotoxicity and that saturated fats increase the susceptibility of patients to NAFLD and NASH progression [19]. Yamada et al. [28] found that the C18:0/C16:0 ratio was the most important correlate of steatosis score. This is in agreement with Roumans et al. [29] that saturated fatty acids may be a marker of de novo lipogenesis contributing to hepatic steatosis.

Individual FA (16:0, 16:1, 20:4, 20:5) concentrations determined by chromatographic analysis can be used to calculate the ratios of f_SI_, f_UI_ and f_PUI_ fractions. One study documented f_SI_, f_UI_ and f_PUI_ fraction ratios of 100/6/5 in NASH patients and 100/14/12 in controls [25]. This is in agreement with our data obtained by MR. Using a diversity of methods and patient groups, we documented a ratio of 100/4/2 in patients with post-LT NASH compared to 100/12/6 in non-steatosis recipients.

In LT candidates (WL group), hepatic lipid saturation increased while unsaturation and polyunsaturation decreased in correlation with increased HFC. However, differences between steatotic grades were less pronounced than in the Tx group. The presence of cirrhosis may have influenced FA profiles and liver metabolic activity. HFC typically decreases with cirrhosis development even in patients with NASH cirrhosis. Accordingly, only two of our WL patients exhibited steatosis grade 2 or 3. We speculate that FA profiles were not significantly affected by factors such as the presence of cirrhosis.

MRS holds a number of advantages over biopsy in that it is non-invasive and generates continuous values, unlike semi-quantitative histological evaluation. Fat content is also measured over a larger liver area, with total liver fat content typically greater than that produced from a small liver biopsy sample. As expected, histological steatosis grade correlated with liver fat content based on FF parameters determined by MRS in the Tx group. Previously, we repeatedly confirmed that liver graft fat content measured by in vivo MR spectroscopy correlates with steatosis grade determined by histology and that this correlation is not linear [14].

Due to low spectral resolution, individual fatty acids could not be characterized. Only hydrogen atoms in functional groups representing FA were determined. Therefore, relative signal intensities were not corrected for individual T2 relaxation times (only 27 ms for water and 60 ms for lipids). To obtain a more accurate T2 set, individual signals would have been better separated using a more suitable solution. This is made difficult due to the overlapping signals in mixtures of different fatty acids. A detailed analysis of high-resolution fatty acid spectra in the database [30] shows that the range of chemical shifts in some protons can be much greater than that used to characterize functional groups in vivo. Conformational changes in FA chains can also affect the chemical shifts of hydrogen groups. All of these factors result in broad signals. Therefore, calculations based on in vivo spectra should only be considered semi-quantitative given that we used constant T2 values for this group of patients.

Our study also lacked a sufficient number of patients in some groups and we were unable to obtain biopsy results in the group of patients before LT for ethical reasons.

## 4. Materials and Methods

### 4.1. Subjects

We performed 47 liver MR examinations in 47 LT candidates (WL group) and 101 examinations in 94 transplant recipients (Tx group; 6 months–20.6 years after LT) between the years 2015 and 2019. Seven patients were examined twice (6 and 12 months after LT). Clinical and laboratory data for Tx and WL groups are given in Table 4.

All study participants underwent clinical, MR imaging, spectroscopy and laboratory examinations. Liver biopsy was performed in the Tx group according to local protocol. Steatosis was classified based on four grades using a scale by Kleiner [22]: S0 in <5%; S1 in 5–33%; S2 in 33.1–66%; and S3 in >66% of affected hepatocytes. This histological grading correlated with the MRS grading calculated from FF values (ranges for LT recipients [14]: S0^mr^ for FF < 0.81; S1^mr^ for FF = 0.81–6.07; S2^mr^ for FF = 6.08–15.9; and S3^mr^ for FF > 15.9). The NAFLD activity score (NAS) was calculated [22] based on scores for histological steatosis (0–3), lobular inflammation (0–3) and ballooning (0–2). NASH was defined as NAS ≥ 5.

The experimental protocol was approved by the ethics committee of the Institute for Clinical and Experimental Medicine and Thomayer Hospital according to the Declaration of Helsinki. All subjects provided their written informed consent prior to participation in the study.

All LTs were performed at the Transplant Center of the Institute for Clinical and Experimental Medicine, Prague, or at the Transplant Center of the Center for Cardiac and Transplantation Surgery, Brno, from cadaveric organ donors registered at the Czech Transplantation Coordination Center database. No organs from executed prisoners were used.

### 4.2. Phantoms

Phantoms were prepared from a water-based lard emulsion in 4% agar gel [31]. Concentration values of lard weight were 2.5, 5, 10, 15, 20 and 30% (weight). Measurements were taken in a water bath at 37 °C, with lard composition verified by chromatography (saturated fatty acids = 39.4%, monounsaturated fatty acids = 49.2%, polyunsaturated fatty acids = 11.3%).

### 4.3. MR Examination

MR examination involved imaging and spectroscopy, each part taking approximately 60 min to complete. Subjects were examined in a supine position using the Siemens MAGNETOM Trio 3T MR system equipped with an eight-channel surface body array coil.

In addition to standard localizer sequences, T2-weighted HASTE sequences (half-Fourier acquisition single-shot turbo spin-echo sequence) in transversal and coronal anatomic directions (echo time/repetition time TR/TE = 1800/96 ms; 20 slices 10 mm in width) were used to position the volume of interest (VOI) for MRS.

A standard PRESS (point-resolved spectroscopy sequence) was used (TR/TE = 4500/30 ms) to measure HFC. MR images in three basic anatomical orientations were used to set the VOI at 40 × 30 × 25 mm in biopsy position (Figure 1a).

Single breath-holding spectra acquired with water signal suppression were measured twice; spectra without water suppression were repeated three times. To estimate T2 relaxation times, a set of PRESS spectra with echo times of 30, 50, 68, 135, 180 and 270 ms was acquired without water suppression; T1 values were not calculated.

### 4.4. Data Evaluation

MR spectra were evaluated using the LCModel [15]. The basis set included 10 lipid and water signals at 4.7 ppm (Table 1). The Lip53 signal represents the sum of signal intensities at 5.2 and 5.3 ppm.

The total number of spectra was 865. Of those, 520 spectra were without water suppression and 345 spectra with water suppression. After quality control tests of spectra calculated using the LCModel, poor quality spectra were excluded. In total, 441 non-water-suppressed spectra and 279 water-suppressed spectra were used for data analysis. Fat fraction (FF) was calculated according to the following equation using intensities of water signals from non-suppressed spectra and intensities of lipid signals from spectra with suppressed water signals

(1) $$\mathrm{FF}\;\left[\%\right]=100*\frac{I_{F}^{\mathrm{cor}}}{I_{F}^{\mathrm{cor}}+I_{W}^{\mathrm{cor}}}$$

where $I_{F}^{\mathrm{cor}}\;$ is the sum of lipid signal intensities and $I_{W}^{\mathrm{cor}}$ is water intensity including signals at 5.3 ppm; both were corrected for T2 saturation according to the following equation

(2) $$I_{F,W}^{\mathrm{cor}}=I_{F,W}/e^{-\mathrm{TE}/T2}$$

where I_F,W_ are experimental signal intensities, TE is echo time and T2 is relaxation time.

In most patients, we measured T2 values. However, variance of T2 values calculated according to the mono-exponential model was wide, i.e., between 21 and 87 ms for lipids and 19 to 54 ms for water. To compensate, we employed the values used in our previous study, i.e., T2 = 27 ms for water and T2 = 60 ms for lipids [14].

Fractions of hydrogen atoms in saturated (f_SI_), unsaturated (f_UI_) and polyunsaturated (f_PUI_) bonds in all FA chains were determined according to the following equations

f_SI_ = (Lip09 + Lip13 + Lip16 + Lip24)/(Lip09 + Lip13 + Lip16 + Lip21 + Lip24 + Lip28 + Lip53) (3)

f_UI_ = (Lip21/2 + Lip28)/(Lip09 + Lip13 + Lip16 + Lip21 + Lip24 + Lip28 + Lip53) (4)

f_PUI_ = (Lip28)/(Lip09 + Lip13 + Lip16 + Lip21 + Lip24 + Lip28 + Lip53) (5)

These equations are similar to those proposed by Ericson [19] with a few minor changes: (a) Lip53 signal intensities in the denominator; (b) the fraction f_UI_, which represents the number of olefinic protons (based on the linoleic and oleic acid model where the contribution of allyl protons is divided by 2 with the contribution of the bis allyl Lip28 signal).

This modification is consistent with the results of our previous phantom study [31] (Table 5). Phantom analysis proved a better fit in the case of modified Equation (4).

### 4.5. Statistics

Data management was carried out using Microsoft Excel. GraphPad Prism 9 statistical software (www.graphpad.com, accessed on 14 September 2021) was used for data analysis. Univariate analysis comprised the chi-square test, t-test or Mann–Whitney U test. Multivariate analysis consisted of one-way ANOVA with Holm-Sidak’s multiple comparisons test or the non-parametric Kruskal–Wallis test with Dunn’s multiple comparisons test. *p* values < 0.05 were considered statistically significant and used as the confidence interval.

## 5. Conclusions

MR spectroscopy was used to calculate hepatic fat content in LT recipients and candidates, with data on LT recipients subsequently compared with histological findings. We also performed a detailed analysis of ^1^H MR spectra to determine lipid signal profiles. We found that liver fat content, represented by FF values, is adept at distinguishing NASH, NAFL and non-steatotic LT recipients. Relative hepatic lipid saturation increased while unsaturation decreased in correlation with increased hepatic fat content. Finally, relative hepatic lipid saturation increased, while unsaturation decreased in recipients with histologically proven post-LT NASH or NAFL compared to non-steatotic patients. We conclude that MRS provides non-invasive support for NASH diagnosis.

## Figures and Tables

**Table 1:** Chemical shifts and corresponding structures in fatty acid chains in the human liver used for lipid profile calculation. LCModel [15] starting parameters for calculation of signal intensities in liver spectra are also shown.

Signal Label	Ppm	Expected Signals	LCModel Starting Parameters			
			Position [ppm]	Position SD [ppm]	Width Min [ppm]	Width Max [ppm]
Lip09	0.9	–(CH_2_)_n_–C**H**_3_	0.87	0.04	0.25	0.45
Lip13	1.3	–(C**H**_2_)_n_	1.28	0.04	0.02	0.55
Lip16	1.6	–O–CO–CH_2_–C**H**_2_–	1.60	0.02	0.02	0.25
Lip21	2.0–2.1	–CH_2_–CH=CH–C**H**_2_– (allylic)	2.02	0.03	0.05	0.25
Lip24	2.4	–CO–C**H**_2_–CH_2_–	2.23	0.03	0.05	0.25
Lip28	2.8	–CH=CH–C**H**_2_–CH=CH– (diallylic)	2.76	0.03	0.05	0.25
Lip41	4.1	–C**H**_2_–O–CO–R (glycerol)	4.10	0.03	0.20	0.30
Lip43	4.3	–C**H**_2_–O–CO–R (glycerol)	4.30	0.03	0.20	0.30
Lip53	5.2–5.3	>C**H**–O–CO–R (glycerol) –C**H**=C**H**– (olefinic)	5.20 5.33	0.03 0.02	0.02 0.20	0.15 0.40
Water	4.7	H_2_O	4.65	0.05	0.01	0.55

SD—standard deviation, ppm—parts per million.

**Table 2:** Distribution of fatty acid fractions (FF) [%] in liver transplant recipient (Tx) and candidate (WL) groups classified according to histological and MRS steatosis grades. (95% confidence intervals are given in parentheses.).

Histological Grading [22]		S0	S1	S2	S3
**Tx group** ** 84biopsies**	**N**	34	34	9	7
	**Mean FF**	0.98	2.71	14.50	23.32
		(0.71–1.25)	(1.82–3.61)	(7.00–21.99)	(13.65–33.00)
**MRS grading**		**S0^mr^**	**S1^mr^**	**S2^mr^**	**S3^mr^**
		**(FF ≤ 0.81)**	**(0.81 < FF ≤ 6.07)**	**(6.07 < FF ≤ 15.9)**	**(FF > 15.9)**
**Tx group** **101MRS**	**N**	22	62	9	8
	**Mean FF**	0.55	2.22	11.41	26.57
		(0.48–0.62)	(1.83–2.57)	(8.95–13.88)	(19.53–33.60)
**WL group** **47 MRS**	**N**	16	29	1	1
	**Mean FF**	0.60	2.01	*15.23 ^+^*	*20.74 ^+^*
		(0.50–0.69)	(1.30–2.73)		

^+^ Only data for one subject are shown for comparison purposes.

**Table 3:** Fractions of hydrogen atoms in saturated (f_SI_), unsaturated (f_UI_) and polyunsaturated (f_PUI_) functional groups of FA chains in Tx subgroups. Fractions are categorized based on histological results (95% confidence intervals are given in parentheses; N—number of subjects).

	Non-Steatosis	NAFL	NASH	Unknown
**N**	34	39	11	17
**f_SI_ [%]**	77.62	85.36 ^a^	89.31 ^a^	82.00
	(74.87–80.37)	(83.29–87.42)	(88.05–90.57)	(77.36–86.65)
**f_UI_ [%]**	9.24	6.00 ^a^	3.80 ^a^	8.47
	(7.37–11.11)	(4.88–7.12)	(3.15–4.44)	(5.69–11.25)
**f_PUI_ [%]**	4.97	2.86	1.40 ^a^	4.60
	(3.46–6.47)	(2.01–3.71)	(0.92–1.87)	(2.28–6.92)
**FF [%]**	0.98 ^b^	4.11 ^b^	20.50 ^b^	2.62
	(0.71–1.26)	(2.39–5.84)	(13.07–27.93)	(1.45–3.80)

^a^ significant difference from the non-steatosis group. ^b^ significant difference between non-steatosis, NAFL and NASH groups.

**Table 4:** Clinical and laboratory data and results of elastography of patients before (WL) and after (Tx) LT classified by steatosis grade (S0^mr^, S1^mr^, S2^mr^, S3^mr^) evaluated by MR spectroscopy. Standard deviations are in parentheses.

	Tx	S0^mr^-Tx	S1^mr^-Tx	S2^mr^-Tx	S3^mr^-Tx	WL	S0^mr^-WL	S1^mr^-WL
**N**	101	22	62	9	8	47	16	29
**Age (yrs)**	56.4	52.1	57.4	56.6	59.4	58	58.8	57.5
	(11.4)	(12.9)	(11.1)	(10.8)	(6.5)	(9.9)	(10.0)	(10.0)
**BMI (kg/m^2^)**	26.4	21.7	26.8	28.6	27.6	26.8	25.1	27.6
	(4.4)	(2.1)	(4.0)	(3.8)	(3.3)	(4.7)	(4.3)	(4.7)
**Waist circumference (cm)**	98.2	83.6	98.3	109.6	108.4	101.7	97.3	104.1
	(13.2)	(8.5)	(11.2)	(8.8)	(12.9)	(13.6)	(11.9)	(13.9)
**Total bilirubin (umol/L)**	15.2	14.8	15.0	14.0	14.4	60.7	78.1	51.7
	(7.8)	(9.4)	(7.2)	(8.8)	(3.9)	(49.3)	(55.6)	(43.9)
**AST (ukat/L)**	0.43	0.45	0.42	0.56	0.54	1.21	1.29	1.17
	(0.22)	(0.27)	(0.16)	(0.52)	(0.1)	(0.78)	(0.91)	(0.72)
**ALT (ukat/L)**	0.56	0.56	0.54	0.69	0.85	0.81	0.77	0.83
	(0.33)	(0.47)	(0.33)	(0.28)	(0.45)	(0.54)	(0.56)	(0.54)
**ALP (ukat/L)**	1.66	1.47	1.69	1.72	1.62	2.82	2.94	2.76
	(0.99)	(0.57)	(1.07)	(1.10)	(0.73)	(2.48)	(1.32)	(2.92)
**GGT (ukat/L)**	0.91	0.67	0.78	1.78	1.75	2.25	2.04	2.37
	(1.58)	(0.95)	(1.25)	(3.55)	(1.06)	(2.70)	(1.94)	(3.04)
**Triglycerides (mmol/L)**	1.55	1.16	1.36	2.25	3.44	1.11	0.92	1.19
	(1.09)	(0.43)	(0.68)	(1.02)	(3.13)	(0.68)	(0.30)	(0.77)
**Cholesterol (mmol/L)**	4.67	4.59	4.60	5.10	4.74	3.96	3.85	4.01
	(0.96)	(0.74)	(0.96)	(0.87)	(0.95)	(1.56)	(1.32)	(1.67)
**LDL chol (mmol/L)**	2.72	2.67	2.69	2.91	2.65	2.61	2.61	2.61
	(0.78)	(0.55)	(0.76)	(0.79)	(1.08)	(1.14)	(1.25)	(1.12)
**HDL chol (mmol/L)**	1.26	1.41	1.26	1.18	1.09	0.80	0.82	0.79
	(0.39)	(0.38)	(0.35)	(0.47)	(0.59)	(0.41)	(0.41)	(0.41)
**Glycaemia (mmol/L)**	6.12	5.37	6.03	8.02	6.29	5.49	5.35	5.57
	(1.87)	(0.67)	(1.62)	(3.03)	(0.78)	(1.33)	(1.43)	(1.29)
**HbA1c (mmol/mol)**	38.04	33.47	37.00	43.44	40.8	32.28	27.50	34.74
	(10.02)	(6.46)	(7.21)	(11.91)	(8.32)	(10.62)	(6.03)	(11.67)
**C-peptide (nmol/L)**	0.90	0.90	0.89	1.25	1.46	1.34	1.39	1.32
	(0.42)	(0.38)	(0.40)	(0.53)	(0.87)	(0.64)	(0.84)	(0.52)
**Fast insulinaemia (uIU/mL)**	7.92	6.76	7.73	12.24	14.59	20.60	15.52	23.31
	(5.32)	(3.15)	(4.16)	(8.31)	(11.73)	(22.16)	(16.99)	(24.31)
**HOMA-insulin resistance**	2.16	1.63	2.07	4.38	4.23	5.50	4.26	6.16
	(1.66)	(0.84)	(1.2)	(2.71)	(3.53)	(6.76)	(6.46)	(6.93)
**QUICKI**	0.357	0.364	0.355	0.325	0.338	0.327	0.338	0.321
	(0.039)	(0.030)	(0.036)	(0.044)	(0.070)	(0.049)	(0.041)	(0.053)
**Elastography (kPa)**	7.1	6.4	7.2	7.5	8.5	35.9	35.1	36.4
	(1.9)	(1.4)	(1.8)	(2.0)	(1.3)	(13.8)	(13.0)	(14.5)
**NAS score (84 subjects)**	1.81	0.16	1.54	4.43	5.13	n/a	n/a	n/a
	(1.87)	(0.36)	(1.17)	(1.68)	(1.17)			

N—number of subjects; BMI—body mass index; ALP—alkaline phosphatase; ALT—alanine aminotransferase; AST—aspartate aminotransferase; GGT—gamma-glutamyl transferase; HbA1c—glycated hemoglobin; HDL—high-density lipoprotein; LDL—low-density lipoprotein; HOMA—homeostatic model assessment; QUICKI—quantitative insulin sensitivity check index; n/a—not available.

**Table 5:** Results of three methods for calculating unsaturated fraction f_UI_ in the phantom according to Equation (4): (a) from signal intensities of olefinic protons (Lip53); (b) using modified Equation (4); (c) original equation from the literature [19].

Fat Concentration in Phantom [%]	(a) f_Lip53_	(b) f_Lip21/2 + Lip28_	(c) f_Lip21 + Lip28_ [19]
**5**	4.1%	4.6%	8.6%
**10**	4.4%	3.8%	7.3%
**15**	4.0%	3.3%	6.3%
**20**	6.9%	4.4%	8.4%
**30**	5.6%	5.2%	9.8%
**Theoretical values ^+^**	4.5	4.5	8.3

^+^ Calculation based on a model of 9 prominent fatty acids in lard samples analyzed by chromatography. For details, see the literature [31].

## Data Availability

The data presented in this study are available on request from the corresponding author. The data are not publicly available due to ethical reasons.
